# New York Odontological Society

**Published:** 1891-07

**Authors:** 

**Affiliations:** New York Odontological Society


### Reports of Society Meetings.

### NEW YORK ODONTOLOGICAL SOCIETY.

A REGULAR meeting of the New York Odontological Society was held Tuesday evening, April 21, 1891, at the Academy of Medicine, No. 17 West Forty-third Street, New York City.

The President, Dr. William H. Dwinelle, in the chair.

### INCIDENTS OF OFFICE PRACTICE AND CASUAL COMMUNICATIONS.

*Dr. J. Bond Littig.*—Mr. President, I would like to present to the society this evening a method for enamelling the faces of all gold crowns. We all know how conspicuous gold crowns are in the mouth, which characteristic has always been very objectionable to me, and I have tried various devices for remedying that difficulty. Since the introduction of glass fillings, I have hit upon a plan which removes almost entirely the unsightly appearance, and I thought it might be interesting to describe how I did it. After fitting the ordinary gold crown to the root I remove the crown and fill it with modelling composition, to keep it from bending. Then, with the ordinary jeweller's saw, I cut out the lower part of the buccal surface of the gold crown, commencing at the cutting-edge, leaving a ring of gold at the top. I then remove the composition and fit a piece of either thin platinum or gold—I prefer the platinum—to the inside of the crown, just to the edges of the opening made with the saw. I always thicken the crown at the cusps with solder, using twenty-carat solder for that purpose, and, holding it over the burner, I solder the little piece of platinum at one end, and then add the glass filling of the proper shade. It depends a great deal upon how this is fused, whether it brings the color out or not; if it is fused too much, it bleaches; if fused too little, the color is not brought out. After wetting the glass with distilled water, I pack it in over the platinum, and with some bibulous paper make it as smooth as possible; then hold it over the alcohol lamp with the pliers. If not plump enough, I repeat the process until I have it contoured to my satisfaction. I have had some of these enamelled crowns wearing in the mouth for six months, and there has been no change. I thought it was a sufficient test to justify me in describing the process.

I have tried another way too,—that of grinding very thin pieces of porcelain from English teeth, or even American teeth, fitting them towards the surface, from the inside, and fusing glass around them. That gives the color a little better, but it is more work. Enamelling by the first method can be done in ten minutes' time, and I think the gentlemen will find, when I pass this crown around, that the improvement in appearance is considerable.

*The President.*—This is a very interesting subject, and refers to a thought that has passed through the minds of almost every dentist,—the wish, at least,—that this could be accomplished.

*Dr. J. Morgan Howe.*—I can testify to the practical and artistic success of this device which Dr. Littig suggested to me some time since, although I have used it in only one case,—one in which a small cap seemed to be about the only thing that could be used to preserve the tooth, without the necessity of destroying the pulp. Posterior and anterior proximal cavities had so weakened the buccal wall of a bicuspid tooth that it broke down, although the pulp was alive. By means of this device, of putting a veneer of color, approximating the color of the teeth, on the buccal face of the cap, I made what was to me a very satisfactory operation; and, as Dr. Littig says, the use of the glass in facing the gold cap did not take over ten minutes' time.

*Dr. S. G. Perry.*—I have been, for a long time, a believer in mallets. I have used the hand mallet, the automatic, the electric, and, during recent years, the Bonwill mechanical mallet, but I have never given up my old love for the automatic, four or five of which I always keep at hand. I have had some changes made in them which I think have been an improvement. I have had vulcanized onto the small end a black rubber "nozzle," which is very smooth and pleasant to use in the hand, and so shaped that the fingers find suitable support in pressing the instrument. The socket ends of all these instruments are made small and tapering, and become, when held like a pen, quite inconvenient, if used for a long time. I have had these nozzles constricted in their centres, so that the fingers, grasping the constricted portion, find support, and there is no chance of slipping. Some may think that the size of the nozzle would become an objection, by rubbing against the cheek, but I have many of my points made long, and get over the difficulty in that way. These instruments were sent to Hood & Reynold's, in Boston, and made over.

One of the first requirements in an automatic mallet, it seems to me, is that there shall be no side movement. I think these are close-fitting, and very smooth in their action. One other advantage is to have the springs made soft and easy to work. Following a suggestion made by Dr. J. A. Woodward, I always anneal the spring before I use it, which makes it very soft. Recently I have had some little burs made for finishing fillings, which are not cut like ordinary burs, being made very fine. They are like a fine file, and they give a finish that I cannot get with anything else, unless it is a fine stone, which wears out rapidly and which cannot be used very small. They do not cut rapidly, but they are not meant to do so. They are particularly well suited for use at the edge of the gum on all labial surfaces.

Some time since I called the attention of this society to some little sand-paper disk mandrels that I had made, with a delicate nut and holder for the same. At that meeting, Dr. Jarvie stated that he had seen, at Albany last year, a mandrel made without a nut. I thought about that, and afterwards remembering that we have mandrels made with corkscrew points for holding leather wheels, etc., I saw very quickly that that same idea, applied to our disks, ought to succeed. I had some of those mandrels made in that way, and, much to my gratification, I found they worked quite perfectly. One would suppose a sand-paper disk would not be strong enough to bear the force, and would tear off, but the sand-paper is flexible and springs away from the tooth or filling while revolving, so that, after all, they do hold well enough to wear out the disk. I have the disks and mandrels made in three different sizes, and find them almost invaluable in finishing fillings. The smallest disks are only one-quarter of an inch in diameter, and they reach where the large ones cannot. These little ones are invaluable in finishing on the labial surface close to the gums and along the exposed borders of proximal fillings.

If the cutting surface is on the outside, they do not slip off; and if it is on the inside, they still do not pull off unless they are used carelessly. When a disk becomes warm, touch it with the finger, and off it goes; there is no time lost in changing a nut. Whatever advantage may arise from a mandrel without a nut the credit is not with me, of course. The suggestion came from Dr. Jarvie. I have never learned the gentleman's name who showed them, and have never heard a definite description of how they are made.[1]

*Dr. Brockway.*—I think that is the way they were made.

---

[1] Since these remarks were made I have learned they were first shown by Dr. M. L. Rowe, Albany, N. Y.—[S. G. P.]

*Dr. Perry.*—The disk-cutters and mandrels were made for me by Lukens & Whittington, of Philadelphia.

I repeat that I want to give this gentleman, whoever he is, the credit which is due him, for from him, through Dr. Jarvie, I got this idea. The small disks and slender mandrels, however, are my own idea, and I again show them, not to claim credit for the manner of attaching the disk, but in order to show what beautiful finishing instruments they are when used without the nut, because, as the corkscrew does not project through the disk, its whole revolving surface can be held against the filling. Of course, the disks have to be specially made. I have them made in three sizes, and I have the three sizes of mandrels. Dr. Northrup asks how large a size can be used in these corkscrew mandrels. I use the largest size of disk sold in the market, and it seems to apply nearly as well as the smaller sizes by having the thread made coarser, and the mandrel larger, but a little more care must be taken in using the large ones, as they sometimes run off and might easily fly back into the throat. Time is saved by their use, but with the large ones, for one who operates with an attendant and can have a supply of disks held by a nut at hand, it may be doubted if there is much gain.

In making these, some very nice points are involved, which, if not regarded, will lead to failure. One must consider the thickness of the disk, its toughness, and the size of the hole, and the mandrel must be of the right size, and the corkscrew thread cut on it must be clean cut, and must increase in diameter with sufficient rapidity to bore its way into the paper or cloth, and yet keep the disk well centred.

*Dr. B. C. Nash.*—I remember to have seen exhibited at the State Dental Society, a year ago, an instrument designed for the same purpose as that shown by Dr. Perry. It was not of the corkscrew shape, but had a small button-like end with a pointed centre and a screw thread cut into it. With this instrument, while revolving, disks were readily picked up and disengaged at will.

*The President.*—As it was desired to give Professor Heitzmann all the time possible for his address at the last meeting, the discussion of the paper entitled "Treatment of Proximate Surfaces," which I read, was deferred until to-night. If there are any remarks to be made upon it, they will be in order at this time.

*Dr. S. E. Davenport.*—I was unable to be present at the last meeting, and therefore did not hear the paper referred to; but as I have had the pleasure of listening on many occasions to our president's views on the treatment of proximate surfaces, I feel sure that I am right in commending the points to which he referred, and his manner of referring to them. This society is to be congratulated on having for its president the discoverer of cohesive gold, and the first to apply it to the restoration of the natural forms of the teeth. Essays from his pen are valuable indeed!

I have felt quite a little regret, ever since the evening Drs. Wilson and Winkler read papers on the same subject, that I did not place myself on record at that time in favor of contour fillings.

Drs. Allan and Jarvie led in the discussion of those papers, and while both gentlemen believe in the principles of contouring, they had most to say that evening in favor of flat gold fillings in the teeth of patients who are unable, either because of ill health or lack of means, to bear the fatigue or expense of large gold contour operations.

It is of such cases that I would speak particularly, and I take exception to the course of reasoning advanced by Drs. Allan and Jarvie, for several reasons.

I presume we all agree that any patient whom we accept is deserving of our best efforts. Why, then, should we deprive the invalid, or the patient with a light purse, of the gum-protecting, decay-preventing contour fillings?

If we accept the principles of contouring in the abstract, is it not quite as necessary that food-crowding should be prevented in the invalid's mouth as in any other, and should we not in honesty make the class of fillings in the teeth of the patient with but moderate means which shall be most likely to prevent the recurrence of decay?

For such cases, then, my creed is: Contour fillings; material to be chosen according to circumstances.

I agree that in such mouths gold contours are usually out of the question; but to me it seems better to change the material than to change the plan.

By the aid of matrices amalgam can be rapidly introduced and the contour accurately restored, a thin layer of gold for the biting surface attached by the Clapp method at the same sitting, or a gold edge or corner added at another sitting, enable us to use the alloys even where the cavity is near the front of the mouth.

In mouths where the zinc-phosphates have been tested and found to be fairly durable, I would contour with that material such teeth as I feared would be made unsightly by amalgam, not that I place much dependence upon the zinc-phosphates, but I am free to confess that I would prefer such fillings well contoured with perhaps a layer of gutta-percha at the cervical margin to flat gold fillings no matter how perfectly made.

I should expect the zinc-phosphate fillings in any mouth not strongly alkaline to protect both teeth and gums better than flat gold fillings, and with less necessity for subsequent operations at or under the margin of the gum.

*Dr. George S. Allan.*—I speak feelingly on this subject, though I should have no reason for so doing, for I never wrote more honestly in my life than I did when I wrote my late paper. I was brought up on hard gold, as it were, on cohesive gold, and I have no idea of the time when I did not believe that the contour filling was the filling to adopt in all practical cases. Now, the question hinges on just this: What are the practical cases? I found, on looking around and watching, as far as possible, the operations of my fellow-dentists, that while every dentist of note and character wished to be put on record as adopting the contour principles, as a matter of fact the bulk of the practice of the day is on the line of the old face filling, and I thought there must be a reason for it.

I also noticed another fact,—that there were certain dentists, like Mr. Dunning, who had to stop work, unfortunately, some years ago, on account of failing eye-sight, who not only ignored the contour fillings, but, very strange to say, whose record for saving teeth was most excellent. In my paper I came out in words which could not be doubted by any one who could understand the English language, that I was a firm believer in the contour method of filling teeth, and that I even pushed things to an extreme, as I now do, to put in contour fillings; but they are not practical at all times. You all know the reasons. There are a great many reasons why the contour filling is not the filling to adopt at all times, in all places, and in all seasons. That paper of mine was misunderstood, and has been misrepresented in print. I am a firm believer in the contour filling system, and I practise according to my convictions.

I do not recollect what I said when Dr. Wilson read his paper. While I fully agree with Dr. Wilson,—and no one recognizes his ability more than I do,—I was perfectly conscious, at the same time, that while he was praising the contour filling and going over the old story with which we are all so familiar, he did not take the position that I wanted him to take, and which I knew he could take, namely, to point out some of the difficulties and how he managed to overcome them. I wanted him to tell us of some details that he had worked out, which would help us in that work. I never intended going on record as favoring face fillings where contour work was possible.

I cannot make it plainer, it seems to me, than I do to-night, that my meaning was misunderstood. Though I acknowledge that I was weary of this incessant ringing of the changes of the benefits of the contour filling, and not one word being given to the value of the face filling.

*Dr. Perry.*—A gentleman hands me this, which I will read:

"In restoring a tooth to its normal condition, if it were possible to replace the original tooth, you would do it, would you not? Of course you would, and so you would be justified in placing there its contoured counterpart."

For a long time I have felt with Dr. Davenport, that in some of these cases, where contours are not practicable with gold, amalgam fillings would be justifiable.

I want to express a strong regret for ever having used copper amalgam. I think it will not only blacken the teeth, but blacken any man's reputation, and I think the sooner that is known and appreciated in the profession, the better it will be. Unfortunately, it is not a trait of human nature to profit by the experience of others. We like to learn for ourselves. I am commencing now to do over many of these fillings, which I put in two years ago. I have been doing them over for some time past. I have not wanted to be hasty in condemning this material, for I have seen many cases that are doing well, but it is too unreliable and we know too little about it to justify its indiscriminate use. Generally, it does not shrink, and it may have an antiseptic effect on the tooth; but unless it be in very delicate fissures in the crowns of back teeth, I do not know of any place where I should use it.

I have one little patient who has, I believe, forty or fifty little seams or imperfect fissures in the grinding surfaces of her bicuspids and molars, which I have recently filled with copper amalgam; but the widest diameter of most of these would be only the size of the minimum burs, and here I expect it to do well. It does not seem to discolor the living part of a tooth so much, but when softened dentine is not removed, it is that which it discolors, and the substance of the dead tooth it will turn as black as midnight in a short time. I feel impelled to make this severe statement in reference to copper amalgam, because only recently a man long in practice, a member of this society, told me he had just commenced its use, and, knowing one must wait for two or three years to really know its effects, I thought it my duty to warn others as I warned him. It causes, first, the discoloration of all dead substances of the tooth; in the next place, it wears away mechanically, evidently, on the surface, and it wastes away, on proximal surfaces, in a manner that suggests chemical solution. When it becomes necessary to remove such a filling, there is a more sensitive condition to contend with than when the cavity was first filled. When used for the purposes of contouring teeth, it takes but a year, or two years at the most, to allow so much waste from the fillings that food easily passes in between them, and the fillings have to be replaced.

*Dr. Littig.*—Have you found the same result with reference to buccal cavities?

*Dr. Perry.*—I think they would bear it better than other surfaces, because they are more exposed. I have seen some good results from its use, but it is unreliable, and I don't think we yet understand the conditions that will make it a success. It is nasty stuff anyhow, and I think we had better use it more sparingly than we have done.

*Dr. Howe.*—I am very glad that Dr. Perry has spoken on this subject. I have not used copper amalgam for over a year, and during that time I have spoken to several of my friends, expressing an unfavorable opinion of it. They have either replied, "You use it too stiff," or else, "You use it with too much mercury in it," or, "You do not mix it right." I have had some results which coincided with the favorable reports that have been made of it, but they were so few, as compared with the relatively large number of unfavorable results, that I do not consider it at all reliable. Its antiseptic properties have been dwelt upon very greatly, and what has been said has, no doubt, given many dentists the confidence they have in it. I suppose it has been proven to be a permanent antiseptic material, but I find that teeth will decay right alongside of fillings made with it. Zinc-phosphate fillings would seem to me to be more reliable, when they are used with judgment and the limitations of the material are recognized.

*Dr. Allan.*—I think Dr. Davenport hit the key-note. There are a great many cases where a good amalgam filling is vastly better than a face filling. I do not think that an oxyphosphate filling has substance, solidity, and strength enough to stand as a contour filling; but certainly amalgam oftentimes acts admirably. One of the difficulties we encounter arises from the fact that sufficient care is not taken to pack the amalgam solidly from the foundation. It will take time to put in a good amalgam filling. The rubber has to be applied with much care, the cavity shaped, and the amalgam packed, not in a huge mass, but in a dry powder, with as little mercury as possible, and built up pellet by pellet. The rubber should not be pulled off, as where gold has been used, but be cut away with the greatest care, so as not to take the risk of displacing any of the amalgam at the neck of the tooth.

*Dr. George Evans.*—I think it is universally acknowledged by the profession that contour work is the proper form; but when these papers are discussed, this line of argument is generally taken: "As a rule, contour work is the best," always leaving the impression that there are cases in which it is not practicable. Those are the cases which we want to get at. If the gentlemen would present a list of conditions in which contour work would not be advisable, I think we would very quickly get at what we aim.

*Dr. Allan.*—Two weeks ago, last Sunday, I sadly looked upon the face of one of my oldest and dearest friends, as he lay in his coffin, in the house that he had occupied ever since he came to New York. As a boy I knew him in Cleveland, and probably there are few here who knew him as intimately and through as many years as I have, who have known both his social and professional life. I allude, as you all know, to our late friend, Wm. H. Atkinson, a man brought up under many disadvantages (he was born in the wilds of Western Pennsylvania) and in the greatest poverty, but by the force of his intellect, and his intellect only, for he never had the chance while a boy of acquiring an education, he had made himself a name which was known the world over.

No man ever lived who worked more fully, heartily, earnestly for his profession than Dr. Atkinson. Whatever may have been his faults, he was true to his profession and true to his brother practitioners. I think it but right, then,—in fact, it is our bounden duty,—to take some fitting action, while his memory is yet green with us, to put upon record what we, as a society, think and feel, and wish others to think and feel with us, respecting Dr. Atkinson.

I therefore call upon the chair, with the permission of the Society, or its acquiescence, to appoint a committee of three to take proper action in this matter.

*The President.*—The chair appoints as that committee, Drs. George S. Allan, Benjamin Lord, and A. L. Northrup, the report to be made at the next meeting.

*Dr. Northrup.*—I beg to move that the president of the Society be added to the committee.

Motion carried.

*The President.*—Since last we met, a prophet has fallen from our midst, and we are left bereaved and desolate.

We have met with a loss that can never be repaired.

There was but one Atkinson, and there can never be another.

He was a man of rare acquirements and rare ability of expression; at times he seemed to us like one *inspired!*

It is a matter of congratulation to us that his *last* effort was here, at the last meeting of our Society, when he reviewed the lecture of his friend Heitzmann before us.

The work he instituted here will go on, and though he be dead, yet shall he *speak* to us continually.

Let it be our duty to perpetuate his memory in this *direction.*

I understand that a movement is already on foot to this end, to, in some way, institute an *Atkinson Educational Memorial*, which I am sure will meet with the approval of all who knew him.

> " He was a man, take him for all in all,
> We shall not look upon his like again."

The essayist of the evening, G. L. Curtis, M.D., D.D.S., was then introduced, who read a paper entitled "A Cursory Glance at Tetanus."

(For Dr. Curtis's paper, see page 438.)

*Dr. Northrup.*—I move a vote of thanks to Dr. Curtis for his very able paper read before us this evening.

Carried.

*The President.*—The subject of Dr. Curtis's paper is now open for discussion.

*Dr. George W. Weld.*—I would like to ask Dr. Curtis whether the bacillus, such as he described, has been isolated.

*Dr. Curtis.*—It has. I have been able to secure the bacillus in a case of tetanus, and have seen it through the microscope, and it has been isolated by many others. Physicians say that some times the stretching of the principal nerve is beneficial where a person has tetanus. It seems to me that there is a little difficulty just here. If the bacillus peculiar to tetanus has been recognized, it does not seem to me that the stretching of the nerve would have anything to do with relieving the patient.

*Dr. Perry.*—I would like to ask Dr. Curtis if I understood him that cases of tetanus have occurred from the replanting of teeth since the modern methods were applied, which of course are antiseptic.

*Dr. Curtis.*—I have stated that cases have been observed in transplanting teeth, not in replanting them. That was about twelve years ago, in Philadelphia. A dentist there transplanted a tooth, and the patient died of tetanus. There are other cases on record, I believe.

*Dr. Northrup.*—Was there not some question whether that case you refer to was tetanus or not?

*Dr. Curtis.*—I have not seen any written statement. I got my information from Dr. Garretson.

*Dr. Perry.*—I have heard repeatedly that it was not by any means an undisputed case of tetanus.

Adjourned.

S. E. Davenport, D.D.S, M.D.S.,
*Editor New York Odontological Society.*

